# Overlapping protein-coding genes in human genome and their coincidental expression in tissues

**DOI:** 10.1038/s41598-019-49802-w

**Published:** 2019-09-16

**Authors:** Chao-Hsin Chen, Chao-Yu Pan, Wen-chang Lin

**Affiliations:** 1 Institute of Biomedical Sciences, Academia Sinica, Taipei, Taiwan R.O.C.; 2 Institute of Biomedical Informatics, National Yang-Ming University, Taipei, Taiwan R.O.C.

**Keywords:** Bioinformatics, Sequence annotation

## Abstract

The completion of human genome sequences and the advancement of next-generation sequencing technologies have engendered a clear understanding of all human genes. Overlapping genes are usually observed in compact genomes, such as those of bacteria and viruses. Notably, overlapping protein-coding genes do exist in human genome sequences. Accordingly, we used the current Ensembl gene annotations to identify overlapping human protein-coding genes. We analysed 19,200 well-annotated protein-coding genes and determined that 4,951 protein-coding genes overlapped with their adjacent genes. Approximately a quarter of all human protein-coding genes were overlapping genes. We observed different clusters of overlapping protein-coding genes, ranging from two genes (paired overlapping genes) to 22 genes. We also divided the paired overlapping protein-coding gene groups into four subtypes. We found that the divergent overlapping gene subtype had a stronger expression association than did the subtypes of 5ʹ-tandem overlapping and 3ʹ-tandem overlapping genes. The majority of paired overlapping genes exhibited comparable coincidental tissue expression profiles; however, a few overlapping gene pairs displayed distinctive tissue expression association patterns. In summary, we have carefully examined the genomic features and distributions about human overlapping protein-coding genes and found coincidental expression in tissues for most overlapping protein-coding genes.

## Introduction

Genome sequences are blueprints of living organisms and play crucial roles in the origination of all life forms. Protein-coding genes are essential elements concealed within genomes to execute cellular functions and biological activities. Among the 40,000 initially predicted human protein-coding genes, currently, approximately 20,000 are comprehensively defined in different chromosome loci within the human genome through repeated rigorous annotations^1,2^. With the rapid accumulation of a considerable amount of next-generation sequencing (NGS) information, researchers can accurately determine protein-coding gene structures and boundaries as well as their isoform expression profiles^3^. This information can enable researchers to obtain clear and updated information on overlapping protein-coding genes in the human genome. Overlapping genes are defined as chromosomal locations of two adjacent gene loci overlapping partially or entirely with each other by sharing a common genomic region^4^. Although it is widely accepted that overlapping genes are common in virus and bacterial genomes to enable compacted genome composition and efficient gene expression modulation^5^, only limited reports exist regarding overlapping protein-coding genes in the human and other mammalian genomes.

A previous systematic analysis of human overlapping genes revealed that approximately 10% of the human protein-coding genes overlap^6^. However, previous studies on overlapping genes have encountered major challenges regarding the annotation of natural antisense transcripts (NATs)^7^. This is because non-coding antisense transcripts are increasingly found in genomes with the advancement of NGS platforms^4,8–10^. Thus, the literature contains contradictory findings. For example, Nakayama *et al*.^11^ reported that same-strand overlap events are more common than opposite-strand overlap events, whereas Sanna *et al*.^6^ indicated that different-strand overlapping genes are the major type in the human genome. This points out that careful annotations and utilisations of DNA loci and RNA transcript information on all protein-coding genes might be crucial in cautiously defining overlapped gene pairs and subsequent analyses^12^. In this report, we mainly investigated the well-annotated overlapping protein-coding genes in the human genome. The NATs within the gene loci could have modulated the steady expression level of gene transcripts through antisense RNAi mechanism^13,14^. Thus, it is essential to use comprehensively annotated gene transcript information and better coverage NGS datasets in order to carefully address the NAT expression modulations on the host protein-coding genes. It is still a challenging mission to establish comprehensive alternative-spliced gene transcript repertoire for such analysis due to the current short read based NGS platforms. Therefore, with current updated human genome assembly and well-annotated protein-coding gene information^15^, we are more interested to interrogate the expression relations of overlapping protein-coding gene pairs at their gene level using available gene level expression datasets. Nearby protein-coding genes could have particular expression modulations due to their chromosome location proximities as well as feedback biochemical interactions between their protein products. There are no recent reports on the protein-coding and protein-coding gene pairs with the available large NGS datasets. In this study, we specifically examined the gene architectures and gene expressions on human overlapping protein-coding genes.

Regulations on adjacent overlapping gene transcripts constitute an appealing topic. Overlapping gene loci or neighbouring gene loci on chromosomes could under analogous global transcription control owing to their shared chromatin domains or compartments. This phenomenon is more evident in the tissue specific gene expression modulation during differentiation and development. When the compacted chromatin domains opened for transcription activities in cells, adjacent or neighbouring gene loci are subjected to the recognition of transcription complex simultaneously^16^. It is reasonable that overlapping protein-coding genes would show coincidental expression patterns. There are reports that co-expression and co-regulation patterns found within such neighbouring genes, which also were grouped as gene clusters^17–19^. Evolutional conservation of these overlapping or adjacent genes were reported not only on their chromosome positions, but also their co-expression patterns^20,21^. However, it is also true that fine modulations or other post-transcriptional regulations would still occur inside the cell with respect to the individual genes between overlapping protein-coding genes. In general, the coincidental expression pattern of overlapping protein-coding genes is commonly recognized. It is also likely that the transcription of nearby overlapping genes could have synergistic or antagonistic modulations^22^. For example, the expression of the *MYCN* gene is coregulated with that of its paired overlapping gene, namely *MYCNOS*^23^. By contrast, the transcriptions of most nested overlapping genes in the human genome are inversely correlated^24^. Furthermore, some of the overlapping genes are tissue-specific^24^. Zhou *et al*.^25^ also observed *VLCAD* and *DLG4* to be paired overlapping genes whose mRNA expression profiles varied in different tissues, indicating tissue-specific transcription controls in certain overlapping gene pairs. Accordingly, these overlapping genes can be independently regulated. These inconsistent results suggest the need for conducting additional investigations on the transcriptional expression and promoter regulations of overlapping genes by using NGS data.

## Results

### Overlapping protein-coding genes

**Figure 1:**
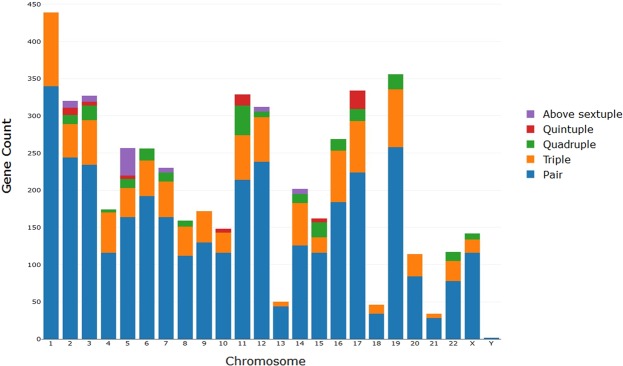
Numbers of overlapping genes according to chromosome positions. Five types of overlapping gene groups were noted: paired, triple, quadruple, quintuple, and above sextuple. Chromosomal distributions of all five overlapping gene groups are displayed.

We used 19,220 protein-coding gene records to investigate overlapping protein-coding genes. With the advancement of the NGS platform, researchers can now use more comprehensive information on gene annotations and transcriptome data and apply the well-maintained Ensembl gene annotations. On the basis of a simple criterion based on shared/overlapped genomic regions, we found 4,951 human protein-coding genes to overlap in terms of their physical gene boundaries (Supplementary Table 1). Thus, approximately one-quarter of all annotated human protein-coding genes were determined to overlap. Among the 4,951 genes, 71.9% were paired overlapping genes and 20.48% were triple overlapping genes. As illustrated in Fig. 1, we observed that the paired overlapping genes constituted the most common type of overlapping genes on all chromosomes. In addition, approximately 4.77% of the total overlapping genes were quadruple overlapping genes, and these genes were mostly on chromosome 11. Quintuple and above sextuple overlapping genes constituted only 1.41% and 1.47% of the total overlapping genes, which were distributed on seven and six different chromosomes, respectively. Chromosome 17 had the highest number of quintuple overlapping genes (25 of the 70 quintuple overlapping genes). Notably, in the aforementioned sextuple genes, two protocadherin gene clusters on chromosome 5 were the largest overlapping gene groups (22 and 15 overlapping genes in each cluster) (Fig. 1).

### Paired overlapping protein-coding genes

As mentioned, of all overlapping gene types, the paired overlapping genes constituted the highest proportion of the genes on all chromosomes. Moreover, the expression of the paired overlapping genes may be directly affected by the nearby overlapping partners. Hence, to elucidate the overlapping gene structures and their gene expression associations, we further investigated the expression of the paired overlapping genes in cancer cell lines. We identified 3,558 paired overlapping genes (1,779 pairs) from 19,220 protein-coding genes. The shortest overlapping gene measured 176 bp, and the smallest overlapping block measured 579 bp (Table 1 and Supplementary Fig. 1). By contrast, the longest overlapping gene measured 1,987,245 bp, and the largest overlapping block measured 2,071,405 bp. As presented in Table 1, the mean gene length of Gene_F (84,594 bp) was more than that of Gene_L (51,411 bp). Among the 3,558 paired overlapping genes, 421 were embedded genes, which had no overlapping intervals (Supplementary Table 2). Notably, we observed cases of extreme proximity between these overlapping gene pairs and adjacent genes (1 bp for Distance_F and 3 bp for Distance_L; Table 1). These might be classified as triple overlapping gene groups if their gene boundary annotations were changed with updated annotations.

**Table 1:** Basic information of paired overlapping genes.

	Min.	Max.	Mean ± SD
Gene Length	176	1987245	68002 ± 129004.7
Gene_F	392	1825171	84594 ± 151411.4
Gene_L	176	1987245	51411 ± 99176.9
Overlapping Interval	0	284372	9343 ± 19234.9
Block Length	579	2071405	109128 ± 159514
Distance_F	1	4088861	69091 ± 227137.9
Distance_L	3	22512734	94364 ± 591428.2
Average overlapping Interval (by each Chromosome)	4497	37722	9344 ± 2877.2

Note:

Gene_F: Frontal gene of paired overlapping genes.

Gene_L: Lateral gene of paired overlapping genes.

Overlapping interval: Overlapped regions of Gene_F and Gene_L.

Block length: Length from the start position of the frontal gene to the end position of the lateral gene.

Distance_F: Distance between the up-stream gene and Gene_F.

Distance_L: Distance between Gene_L and the down-stream gene.

### Chromosome distribution of paired overlapping gene subtypes

As mentioned, the paired overlapping genes constituted over 70% of all overlapping genes (3,558/4,951) and approximately 18.5% of the total protein-coding genes (3,558/19,220). We further examined the distribution of the four subtypes of the paired overlapping genes on chromosomes (5ʹ-tandem overlap; convergent overlap; divergent overlap; and 3ʹ-tandem overlap; Table 2). The convergent and divergent overlap subtypes contained significantly higher numbers of genes compared with the 5ʹ-tandem overlap and 3ʹ-tandem overlap subtypes (more than 5-fold, 2,980 vs. 578). Although chromosome 1 had the highest number of overlapping genes, the overlapping genes constituted approximately 17.04% of all chromosome 1 genes. Conversely, chromosome 12 had the highest percentage of paired overlapping genes (23.82%), and chromosome Y had the lowest percentage of paired overlapping genes (4.44%), namely only 2 of 45 genes (Table 2). The average gene length, average block length, and average overlapping intervals observed for each chromosome are illustrated in Supplementary Fig. 2. Notably, the paired overlapping genes on chromosome 13 had the longest length and block length, and the paired overlapping genes on chromosome Y had the longest overlapping intervals. Supplementary Table 2 presents the overlapping interval regions in terms of the length percentile of Gene_L (lateral genes). The majority of the overlapping intervals were less than 10% of the lateral gene length (n = 799), especially in the convergent and divergent overlap subtypes. However, 421 genes were completely embedded inside their pair partner genes (Gene_F). Additionally, the 5ʹ-tandem and 3ʹ-tandem overlap subtypes had the highest proportions of 100% embedded gene pairs (51 of 140 genes and 57 of 149 genes, respectively) (Supplementary Table 2). Notably, many of these overlapping genes were also in proximity with their neighbouring genes. Distance_F was clustered within 200 bp and Distance_L was clustered within 150 bp (Supplementary Fig. 3).

**Table 2:** Chromosome distribution of paired overlapping genes.

Chromo-some	5ʹ-tandem overlapping	Convergent overlapping	Divergent overlapping	3ʹ-tandem overlapping	Sub-Total	Total Genes in each chromosome	Overlapping gene % in chromosome
1	26	176	110	28	340	1995	17.04%
2	26	104	102	12	244	1209	20.18%
3	18	102	92	22	234	1039	22.52%
4	10	54	36	16	116	742	15.63%
5	14	70	70	10	164	852	19.25%
6	10	102	66	14	192	1007	19.07%
7	16	70	50	28	164	874	18.76%
8	4	50	52	6	112	656	17.07%
9	6	64	50	10	130	753	17.26%
10	6	62	42	6	116	712	16.29%
11	12	100	80	22	214	1267	16.89%
12	20	120	78	20	238	999	23.82%
13	8	24	12	0	44	313	14.06%
14	10	60	48	8	126	590	21.36%
15	8	68	34	6	116	572	20.28%
16	16	104	62	2	184	812	22.66%
17	8	94	94	28	224	1138	19.68%
18	6	18	10	0	34	263	12.93%
19	24	98	108	28	258	1383	18.66%
20	4	48	24	8	84	523	16.06%
21	4	14	8	2	28	219	12.79%
22	8	32	28	10	78	425	18.35%
X	14	38	52	12	116	832	13.94%
Y	2	0	0	0	2	45	4.44%
Total	280	1672	1308	298	3558	19220	18.51%

### RNA-Seq expression data for paired overlapping genes

To examine the expression levels of the paired overlapping genes, we used expression information from the CCLE database. We cross-checked and matched the identified overlapping gene IDs with the obtained CCLE database information. Subsequently, we retrieved 1,646 overlapping gene pairs (3,292 genes) with CCLE RNA-Seq data for further analysis. For comparison, we also randomly selected the same number of non-overlapping genes as the control group and retrieved their RNA-Seq expression information from the CCLE dataset. In brief, comparing the mean gene length of the control genes and paired overlapping genes revealed that the control group (66,904 bp) had a slightly shorter mean length than did the overlapping group (70,821 bp). The two groups had a similar overall gene expression distribution (Supplementary Fig. 4). Notably, the mean value of RPKM gene expression in the control group was higher than that in the overlapping group (4.6277 vs. 3.2973), which was significant (Mann–Whitney U test, *p* < 0.001). For all human protein-coding genes, the RPKM expression value is 3.7278. Interestingly, the top KEGG pathway enriched for these overlapping protein-coding genes is metabolic pathway, which would imply many of these genes possess house-keeping gene nature.

**Figure 2:**
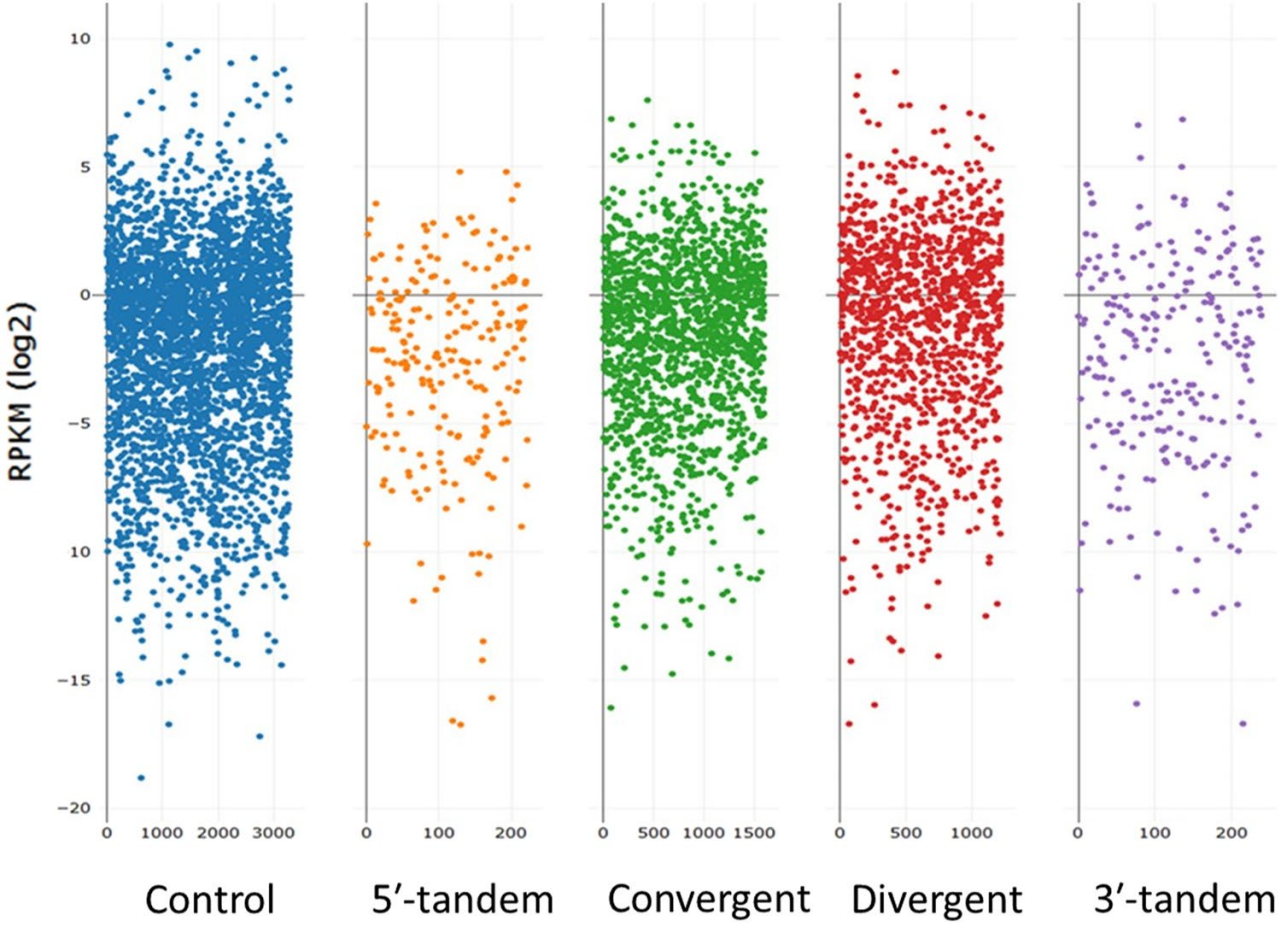
RPKM distribution of the control group and four subtypes of paired overlapping genes. These scatter plots show the RPKM expression levels of the randomly selected non-overlapping gene group (control) and the four subtypes of paired overlapping protein-coding genes: 5ʹ-tandem overlap subtype; convergent overlap subtype; divergent overlap subtype; and 3ʹ-tandem overlap subtype.

We also examined the gene expression patterns in the four subtypes of paired overlapping genes. As expected, we noted that the paired overlapping genes of the convergent and divergent overlap subtypes had a greater number of genes than did those of the other subtypes, as illustrated by the scatter plot (Fig. 2). The Kruskal–Wallis test revealed that the gene expression levels were significantly different between the subtypes (*p* < 0.001). Significant differences existed between all subtypes, except for the 5ʹ-tandem overlap and 3ʹ-tandem overlap comparison (*p* = 0.502), as presented in Table 3.

**Table 3:** Comparison of expression levels of four overlapping gene subtypes.

Comparison of expression values	Statistical *p* -value	FDR *q* -value
5ʹ-tandem – Convergent	<0.001	<0.001
5ʹ-tandem – Divergent	<0.001	<0.001
5ʹ-tandem – 3ʹ-tandem	0.502	0.502
Convergent – Divergent	<0.001	<0.001
Convergent – 3ʹ-tandem	0.003	0.004
Divergent – 3ʹ-tandem	<0.001	<0.001

Kruskal–Wallis test is used to calculate *p* - value.

FDR: False Discovery Rate.

### Association of paired overlapping gene expression

**Figure 3:**
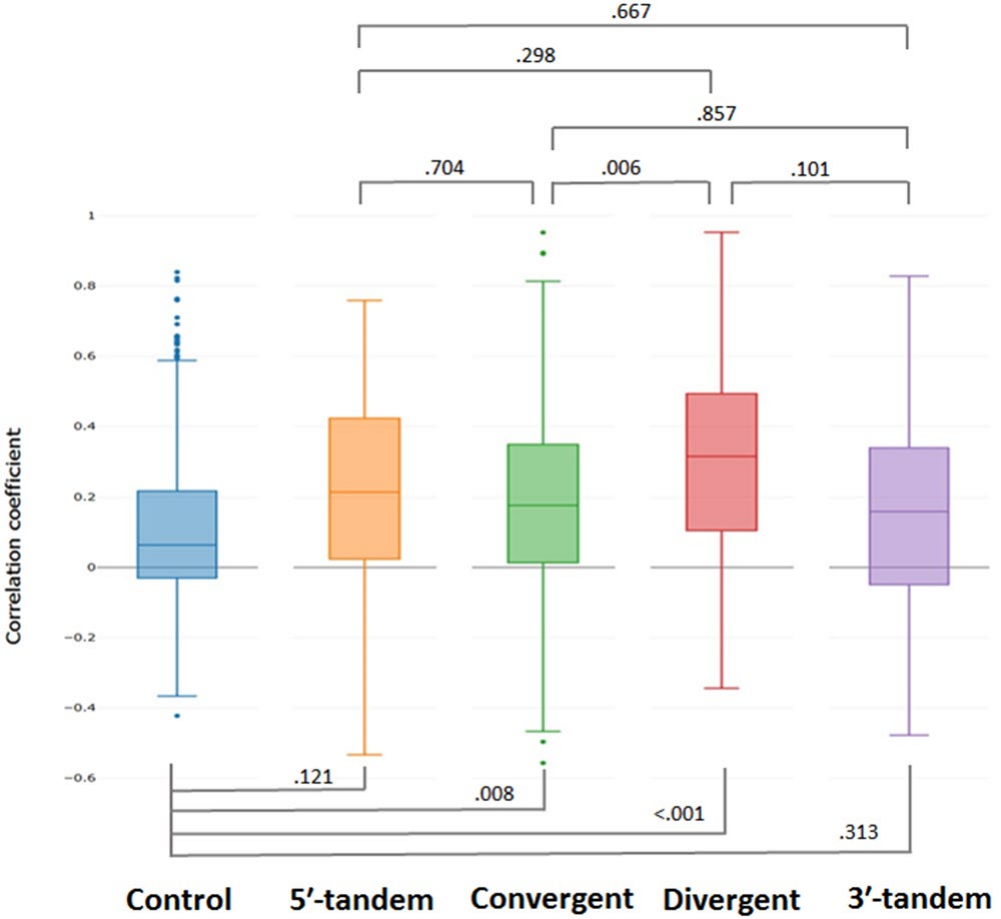
Correlation coefficients of the control group and four subtypes of paired overlapping genes. Boxplots show the correlation coefficient levels of gene expression associations, which included randomly selected non-overlapping genes (the control group) and paired overlapping gene subtypes. Four subtypes of paired overlapping genes: 5ʹ-tandem overlap subtype; convergent overlap subtype; divergent overlap subtype; and 3ʹ-tandem overlap subtype. Fisher’s z test was used to evaluate the significance of differences between two correlation coefficients in subtypes of paired overlapping protein-coding genes. Comparing with the control group, the convergent and divergent overlapping protein-coding gene groups show significant difference. Among the four subtypes, the convergent and divergent overlapping protein-coding gene groups also showed significant variations.

**Figure 4:**
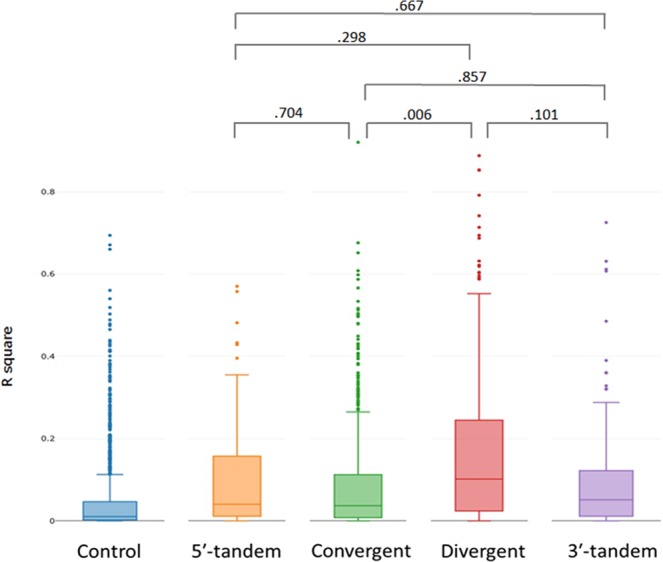
R^2^ values distribution of the control group and four subtypes of paired overlapping genes. Boxplots show the R^2^ values of gene expression associations, which included randomly selected non-overlapping genes (the control group) and paired gene subtypes. Four subtypes of paired overlapping genes: 5ʹ-tandem overlap subtype; convergent overlap subtype; divergent overlap subtype; and 3ʹ-tandem overlap subtype. Fisher’s z test was used to evaluate the significance of differences between R^2^ values in subtypes of paired overlapping protein-coding genes. Among the four subtypes, the convergent and divergent overlapping protein-coding gene groups also showed significant variations.

We conducted correlation and linear regression analyses to test the associations of the expression levels of the paired overlapping genes. As indicated in Fig. 3, the median values of the expression correlations in all subtypes were higher than that in the control group. In addition, the divergent overlap subtype had higher correlations than did the other subtypes, and the 3ʹ-tandem overlap subtype had the lowest median of correlations. This demonstrates that divergent overlapping genes have relatively strong gene expression associations due to the possible common shared promoter regions. Supplementary Table 3 and Fig. 4 present the result of linear regression analysis between gene pairs. We observed statistical differences between the results of the control group and those of the subtypes, signifying that the closest neighbouring genes had the strongest gene expression associations. Among the subtypes of the paired overlapping genes, the divergent overlap subtype exhibited the highest expression associations compared with the remaining subtypes. Specifically, the divergent overlap subtype had more than five times gene pairs have significant associations compared with non-significant association pairs (84% vs. 16%) (Supplementary Table 3). This result corresponds with that in Fig. 4, which indicates that the regression models of the divergent overlap subtype could explain the higher variability of the expression levels compared with the other subtypes and the control group. The convergent overlap subtype had the lowest gene expression association pairs (76%) (Supplementary Table 3). This may be because convergent gene pairs have different promoters, which may eliminate paired gene expression associations. The 5ʹ-tandem overlap and 3ʹ-tandem overlap subtypes had similar proportions of significant expression association paired genes, which were three times the proportions of paired genes with non-significant associations (79% vs. 21% for 5ʹ-tandem overlap and 78% vs. 22% for 3ʹ-tandem overlap) (Supplementary Table 3). For the control group, the proportion of paired genes with significant expression associations was slightly higher than that of those with non-significant expression associations (57% vs. 43%). Accordingly, compared with the control group, all paired protein-coding gene subtypes had higher numbers of significant association pairs; moreover, the divergent overlap subtype had the highest expression associations of gene pairs among the subtypes.

### Tissue expression comparison for paired overlapping genes

**Figure 5:**
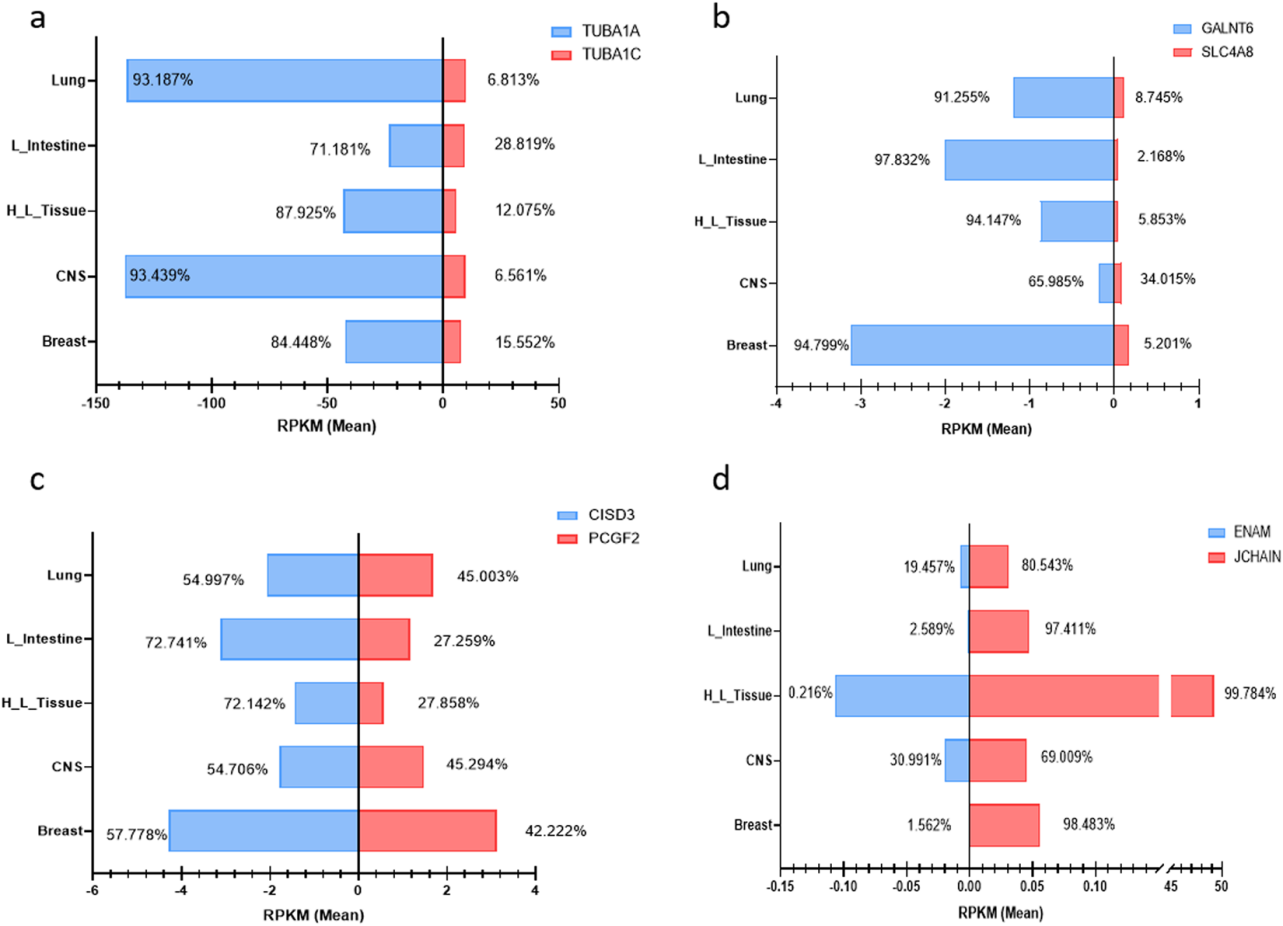
Expression patterns of selected paired overlapping genes in five tissues. The expression of selected paired overlapping genes is illustrated. These gene pairs were selected according to their calculated high coefficient variance values. Both *TUBA1A*/*TUBA1C* and *GALNT6*/*SLC4A8* pairs belonged to the divergent overlap subtype. *CISD3*/*PCGF2* and *ENAM*/*JCHAIN* pairs belonged to the convergent overlap subtype. The four figures: (**a**) *TUBA1A* and *TUBA1C*; (**b**) *GALNT6* and *SLC4A8*; (**c**) *CISD3* and *PCGF2*; (**d**) *ENAM* and *JCHAIN*.

We investigated whether the paired overlapping protein-coding genes had tissue-specific regulated expressions, which had not been clearly examined with a large amount of NGS data. The tissue differences in overall expression levels between the entire group of paired overlapping genes and the control group were non-significant for five tissues (Supplementary Fig. 5). In respective tissues, the paired overlapping group and control group exhibited similar RNA-Seq gene expression patterns. However, the control group seemed to have a slightly higher expression level than did the paired overlapping group for all five tissues, as indicated previously. For most of the paired protein-coding genes, similar expression profiles were found in each pair for all five tissues. We examined the tissue variance among all paired overlapping genes (Supplementary Fig. 6); we noted that the variance values of the paired overlapping genes were small in most of the paired overlapping genes. This implies that there is no tissue expression difference in each pair of overlapping genes. Only less than 1% of the paired overlapping protein-coding genes had more significant variations between tissues (12 out of 1,646 pairs). Interestingly, the convergent overlapping subtype has more gene pairs with higher variance values than other subtypes. This could attribute to the different transcriptional promoters in convergent overlapping protein-coding gene pairs, which may cause the differences in transcriptional modulations. Nevertheless, regarding the individual pairs of overlapping genes, a few paired overlapping genes showed distinct tissue expression patterns. This demonstrates that some specific overlapping genes still had tissue-specific modulations at the transcriptional or posttranscriptional levels (such as miRNA modulation). Several paired overlapping genes showed varied expression profiles in the five tissues (Fig. 5). The expression levels of those overlapping protein-coding genes examined did vary between tissues; for example, *TUBA1A* was highly expressed in lung and central nervous system tissues, and its overlapping partner gene (*TUBA1C*) had similar abundance in all five tissues (Fig. 5a). *JCHAIN* gene is highly expressed in hematopoietic cells, and lowly expressed in other tissue types (Fig. 5d). The *ENAM* gene (partner gene of *JCHAIN*) has very low expression level in all five tissues.

## Discussion

Overlapping genes are essential in genome functions and evolutions. However, they have not been adequately examined in mammalian genomes, including the human genome. The rapid advancements in genome sequencing technologies have engendered a better understanding and characterisation of the physical structures and functional transcription units of human genes. Comparing with previous publications, the numbers of overlapping genes varied in different studies. A possible explanation for this is that gene data extracted from different sources of databases may have been updated throughout the decades. Earlier reports usually contained fewer overlapping protein-coding genes. For example, Veeramachaneni *et al*.^26^ revealed that 4.47% of the genes overlap, whereas Sanna *et al*.^6^ claimed that nearly 13.36% of genes in the human genome overlap^4^. Different data source also affects the analysis outcome. In 2007, Nakayama *et al*. reported 1,692 overlapping genes in human genome with eight different overlapping categories (four on each strand) using NCBI LocusLink data^11^. The numbers of overlapping gene increase to 10,120 by interrogating the mRNA and EST datasets in the EVOG database^27^, which would contain protein-coding genes as well as non-coding genes. In a recent publication by Ning *et al*.^12^, 26% of the human protein-coding genes overlaps, which is similar to our study here (25.8%). Ensembl gene sets were used in both studies (release 85 for Ning *et al*. and release 92 in this study). Importantly, our study here provides more comprehensive genome distribution and gene architect information of overlapping protein-coding genes than other previous studies.

It is also observed that more overlapping gene pairs were found between lncRNAs and protein-coding genes by Ning *et al*. Some of the lncRNAs would be defined as natural antisense transcripts previously, which implied the complexity in genome-wide assessment of overlapping genes. Comparing to previous reports about the expression of overlapping genes, our report here is the first study to demonstrate the overall expression levels in protein-coding genes, which suggests the global chromatin level transcriptional modulation^19^. On the other hand, most of the previous studies mainly focus on the correlation relationship between overlapping genes. Among the different subtypes of overlapping protein-coding genes, our data strongly supports previous findings about the high degree of coregulation on the divergent overlapping group (HH group^22^ or H2H group^12^). The main explanation for high degree of coincidental expression on divergent overlapping protein-coding genes is due to the co-shared promoter regions. The shared promoter regulatory regions would result in the concurrent gene transcription regulation in this overlapping subtype. Interestingly, both previous studies^12,22^ showed embedded subtype of overlapping genes have even stronger correlation in overlapping gene expression. Our data here did not separate the embedded gene pairs in 5’-tandem overlapping and 3’-tandom overlapping groups. It is reasonable to assume that we will have the same findings on the embedded overlapping genes. With currently updated human genome annotations to effectively examine overlapping human genes, our findings imply that the human genome could have a relatively degree of compactness for protein-coding gene regions (overlapping gene clusters) and requires robust gene expression modulations within such selected chromosome regions.

Due to advancement of the NGS platform and availability of considerably high amounts of sequence information, more novel gene transcripts have been identified in addition to overlapping gene transcripts, such as lincRNAs and NATs. Overlapping genes could represent a general phenomenon in de novo gene formation during the evolution process; this phenomenon is commonly observed in rapidly evolving genomes, such as viruses and prokaryotes^28^. By using integrated bioinformatics analysis and multiple NGS datasets (TIF-Seq and Ribosome profiling), Lu *et al*.^28^ reported that more than 4000 putative de novo protein-coding genes existed in yeast genomes and that many of the de novo genes were overlapping gene transcripts carrying novel ORF proteins. This finding implies that genome sequences are highly dynamic in terms of gene transcriptions using alternative promoters and initiation sites than previously understood. In this study, we excluded 433 embedded novel genes (which lack official gene symbols or HGNC names). Those de novo genes might still have critical functions in the human genome and should be explored in the future by using functional genomic approaches. Moreover, different age genes may exhibit complex functional changes, which affect the associations in overlapping genes. Therefore, the evolution of overlapping genes may be crucial. The reason is genes overlap possibly due to chromosome rearrangements so that lead two separate genes to be linked together; alternatively, overlapping genes may result in the generation of a new gene or gene mutation^5,29^. Further experimental research should be undertaken to investigate the functional effects of overlapping genes in humans.

In this study, we also validated that the associations of the expression levels of paired overlapping genes differed significantly from those of randomly selected non-overlapping genes. In addition, we evaluated the associations of the expression levels of four subtypes of paired overlapping genes in major tissues. Previous studies have explored specific overlapping genes or the evolution of overlapping genes^12,25,27,30–32^. Such studies have proposed that the expression levels of overlapping genes could change over time, and such expression associations may influence regulations and functions between partner genes. For example, if the expression levels of overlapping genes are inversely related, this would cause gene function deletion or mutation^13,33,34^. Several studies have revealed that the functional impairment of overlapping genes may be related to diseases or associated with malignant tumours; for instance, *CBS* is related to homocystinuria^35^, *TR* and *COMT* may be associated with schizophrenia^36^ and *CHEK2* may be involved in osteosarcoma^33^. However, the current study examined only the structures and associations of expression levels of overlapping genes, which limits the understanding of overlapping gene outcomes in humans. Thus, overlapping genes may affect human life that should be explored in more detail in future research to obtain clearly understanding of human overlapping genes.

## Conclusion

Research has increasingly shown the existence of overlapping genes in the human genome. We have provided a more updated and comprehensive list of overlapping protein-coding genes. Different types of overlaps of paired genes may involve multiple mechanisms and regulations of gene expression. This study focused on exploring the structures and associations of the expression of paired overlapping genes. We expect this work to provide new insights into overlapping genes in the human genome.

## Methods

### Data source

Human protein-coding annotation data were obtained from the Ensembl release 92—human genes (GRCh38.p12) database. Ensembl release 92 has 64,561 human genes in the GRCh38.p12 assembly, and we retrieved only protein-coding type genes (22,643 records). We first removed 2,773 records with miscellaneous chromosome/scaffold names (assembly exceptions). Of the remaining 19,870 records, 40 had duplicated gene names with different Ensembl stable gene IDs. We therefore removed 20 duplicated records in order to focus on distinct overlapping protein-coding genes. We also removed 107 readthrough transcripts and 433 embedded novel genes (embedded genes without gene description) from subsequent analyses. We further verified the gene records with the NCBI gene2accession file and removed additional 89 records of predicted novel genes without gene description as well as 1 record with a duplicated NCBI gene ID. The final 19,220 records were then used for overlapping protein-coding gene investigations.

For mRNA expression information, we used ‘mRNA expression (RNA-Seq) information’ obtained from the Cancer Cell Line Encyclopaedia (CCLE) database (https://portals.broadinstitute.org/ccle/data, 04-Feb-2018, DepMap_18Q1). The CCLE database contains RNA-Seq data of 1,048 different cancer lines from 26 tissue origins. We selected the expression information of 545 cell lines from the 5 most abundant tissue types (breast, central nervous system, haematopoietic and lymphoid tissue, large intestine, and lung) in this study. The numbers of cell lines for each tissue are outlined as follows: (1) breast: 52 records; (2) central nervous system (CNS): 70 records; (3) haematopoietic and lymphoid tissue (H_L_Tissue): 178 records; (4) large intestine (L_Intestine): 70 records; and (5) lung: 188 records.

### Identification of overlapping gene groups

We defined overlapping genes on the basis of the start and end gene positions on chromosomes. Ensembl database provides updated and comprehensive human gene annotations, which could be used as an excellent and trustworthy resource for gene interrogation studies. The complete gene structure and annotation information were retrieved from Ensembl web database (release 92); and the boundaries of annotated protein-coding genes were defined by the Gene start (bp) and Gene end (bp) fields from Ensembl dataset. Genes overlap if they share a common region. Thus, we identified 4,951 overlapping genes according to this criterion. Subsequently, we divided the overlapping genes into five groups (paired, triple, quadruple, quintuple, and above sextuple), which were based on the number of overlapping genes within a single uninterrupted chromosome region. For the transcriptional modulations of overlapping genes, we further classified paired overlapping genes into four subtypes. This classification was conducted according to the strand-orientation of corresponding genes, commonly used in previous studies. The four subtypes were as follows: (1) 5ʹ-tandem overlap: → →; (2) convergent overlap: → ←; (3) divergent overlap: ← →; and (4) 3ʹ-tandem overlap: ← ←.

### Statistical analysis

R package was used for all statistical analyses and visualisations, which included data preprocessing and descriptive analysis for determining the characteristics and structure of gene data. We also performed inferential analyses, including the Mann–Whitney U test^37^ and Kruskal–Wallis test^38^, to examine the differences in RNA-Seq expression between overlapping and non-overlapping genes. Furthermore, we conducted the Spearman’s rank correlation test^39^ and linear regression analysis^40,41^ to compare the associations of the expression levels of the paired overlapping genes. Statistical significance (α) was set at 0.05. Due to the hypothesis test may cause false positive problems which may misrepresent the results, we then employ Benjamini-Hochberg method^42^ for calculating *q*-vaule to eliminate the number of false positives and analyze the multiple comparisons between Control group and 4 subtypes. Finally, we calculated the variance between the five tissues, which is based on the correlation coefficient of all overlapping gene pairs in each tissue. Variance value is then calculated from the five tissues’ correlation coefficient value for each gene pair. This is to analyse whether tissue-specific expression regulations exist in overlapping protein-coding genes.

## Supplementary information

Supplementary Tables and Figures
