# Supplementary material for: Overlapping protein-coding genes in human genome and their coincidental expression in tissues
Source: Sci Rep. 2019 Sep 16;9:13377. doi: 10.1038/s41598-019-49802-w (PMC6746723; doi:10.1038/s41598-019-49802-w)
Supplement: Supplementary file 1 — Supplementary Tables and Figures [file 41598_2019_49802_MOESM1_ESM.docx]

**Overlapping protein-coding genes in human genome and their coincidental expression in tissues**

Chao-Hsin Chen^1^, Chao-Yu Pan^1, 2^ and Wen-chang Lin^1, 2^

^1^Institute of Biomedical Sciences, Academia Sinica, Taipei, Taiwan, R.O.C.

^2^Institute of Biomedical Informatics, National Yang-Ming University, Taipei, Taiwan, R.O.C.

Corresponding: [wenlin@ibms.sinica.edu.tw](mailto:wenlin@ibms.sinica.edu.tw)

| **Supplementary Table 1.** Numbers of overlapping genes | |
| --- | --- |
| **Overlapping Gene Group** | **Gene Count (%)** |
| Paired | 3558 (71.90%) |
| Triple | 1014 (20.48%) |
| Quadruple | 236 (4.77%) |
| Quintuple | 70 (1.41%) |
| Above sextuple | 73 (1.47%) |
| Total | 4951 (100%) |

**Supplementary Table 2.** Percentile of embedded gene length (overlapping interval) with lateral genes (Gene_L) length

|  | **5’-tandem overlapping** | **Convergent overlapping** | **Divergent overlapping** | **3’-tandem overlapping** | Total numbers |
| --- | --- | --- | --- | --- | --- |
| <10% | 31 | 383 | 347 | 38 | 799 |
| 10-19% | 12 | 112 | 64 | 14 | 202 |
| 20-29% | 7 | 50 | 29 | 5 | 91 |
| 30-39% | 11 | 26 | 16 | 8 | 61 |
| 40-49% | 8 | 28 | 10 | 4 | 50 |
| 50-59% | 4 | 27 | 6 | 3 | 40 |
| 60-69% | 2 | 18 | 6 | 1 | 27 |
| 70-79% | 2 | 17 | 8 | 3 | 30 |
| 80-89% | 4 | 8 | 9 | 2 | 23 |
| 90-99% | 8 | 7 | 6 | 14 | 35 |
| =100% | 51 | 160 | 153 | 57 | 421 |
| Total | 140 | 836 | 654 | 149 | 1779 |

| **Supplementary Table 3.** Significantly associated numbers of gene pairs | | | |
| --- | --- | --- | --- |
| **Subtype** | ***p* < 0.05** | ***p* ≥ 0.05** | **Total Numbers** |
| Control | 942 (57%) | 704(43%) | 1646 |
| 5’-tandem | 88(79%) | 24(21%) | 112 |
| Convergent | 611(76%) | 189(24%) | 800 |
| Divergent | 515(84%) | 99(16%) | 614 |
| 3’-tandem | 94(78%) | 26(22%) | 120 |

**
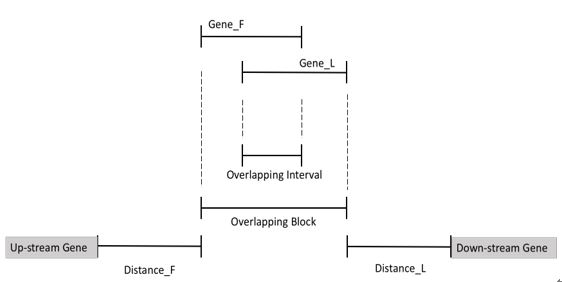
**

**Supplementary Figure 1.** Illustration of paired overlapping gene structures.

Gene_F: Frontal gene of paired overlapping genes. Gene_L: Lateral gene of paired overlapping genes. Overlapping interval: overlapped regions of Gene_F and Gene_L. Overlapping block: length from the start position of the frontal gene to the end position of the lateral gene. Distance_F: distance between the upstream gene and Gene_F. Distance_L: distance between Gene_L and the downstream gene.

**
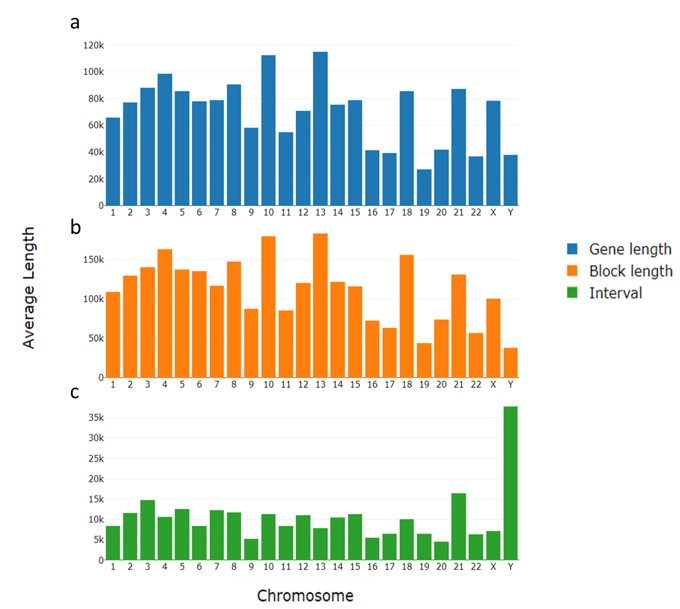
**

**Supplementary Figure 2.** The average length of gene, overlapping block and overlapping interval.

The average length of (a) gene, (b) overlapping block and (c) overlapping interval according to chromosome positions are presented in bar plots.

**
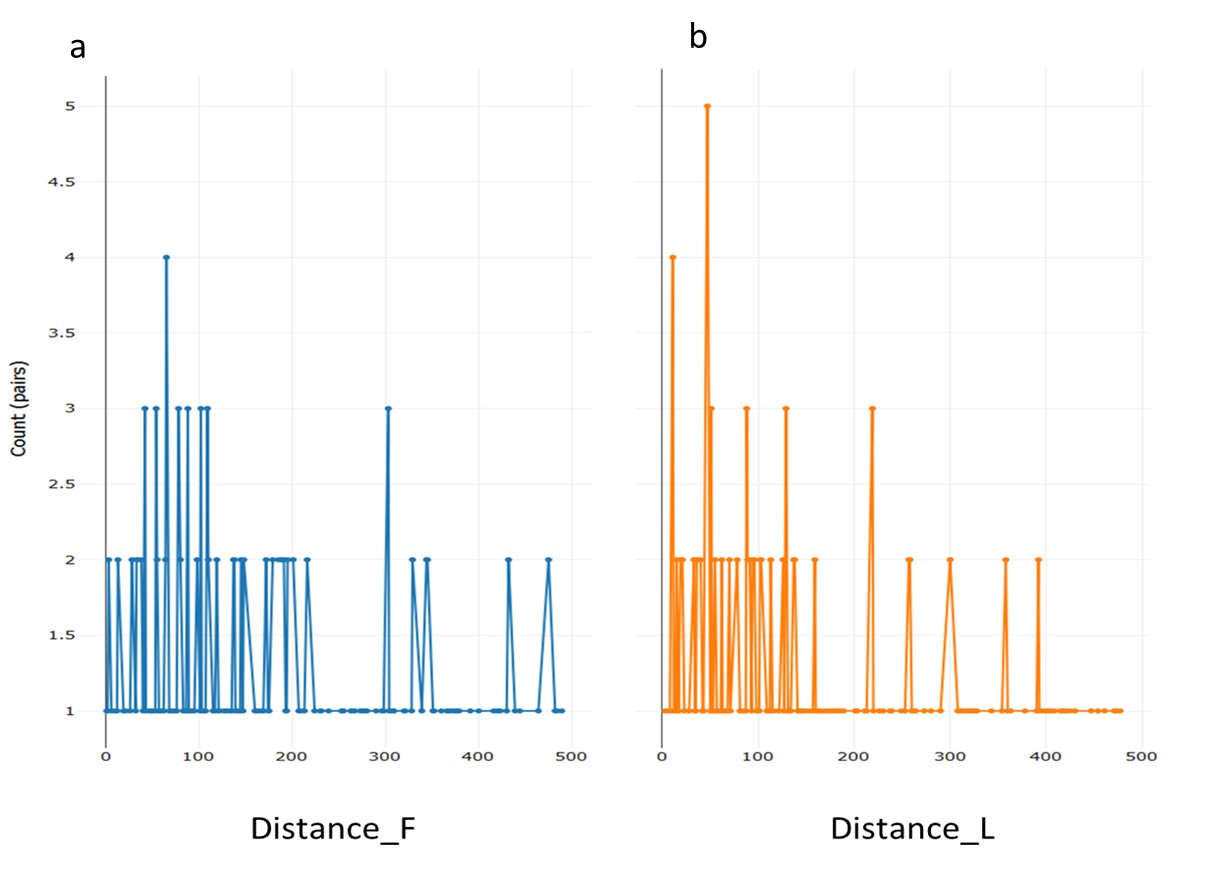
**

**Supplementary Figure 3.** Distances between paired overlapping genes and their neighbouring genes.

We calculated the physical distance between paired overlapping genes and their neighbouring genes in the front and in the back. The figures: (a) Distance between Gene_F (frontal gene) and its neighbouring gene; (b) distance between Gene_L (lateral gene) and its neighbouring gene. Only genes with distance less than 500 bp are displayed.

**
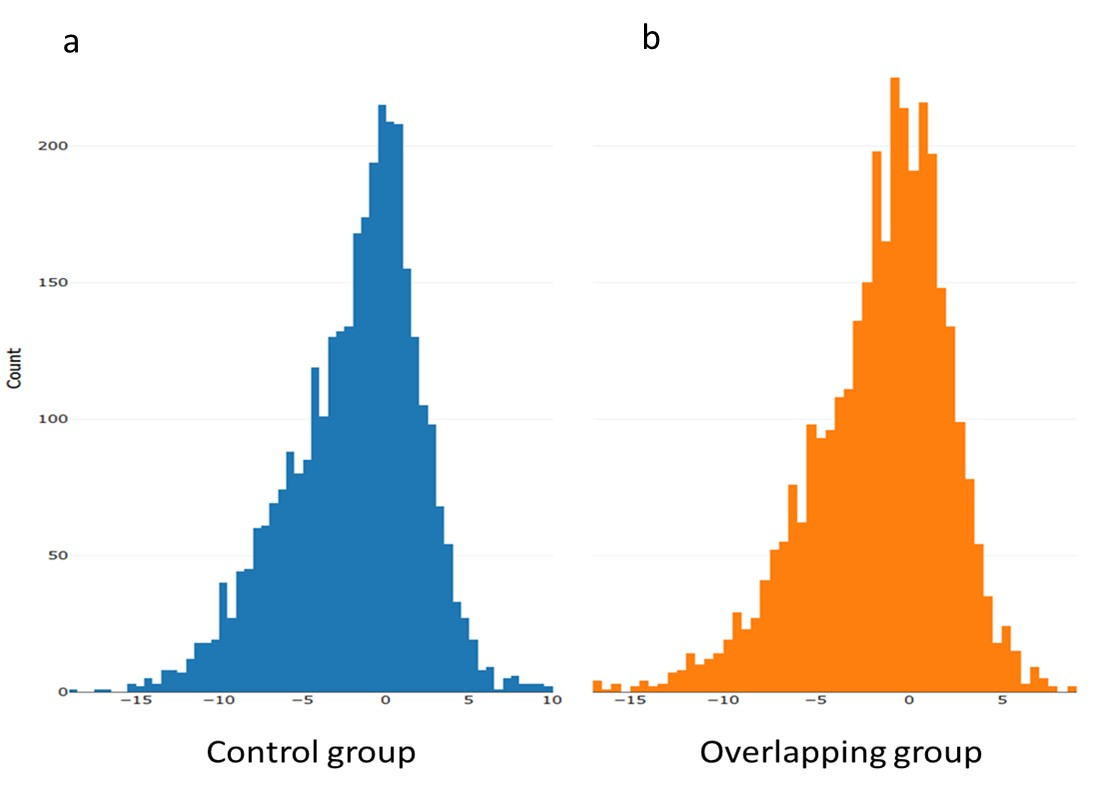
**

**Supplementary Figure 4.** Expression level distribution of non-overlapping genes and paired overlapping genes.

RPKM (log2) distributions for (a) randomly selected non-overlapping gene group and (b) paired overlapping gene group are illustrated in histogram plots.

**
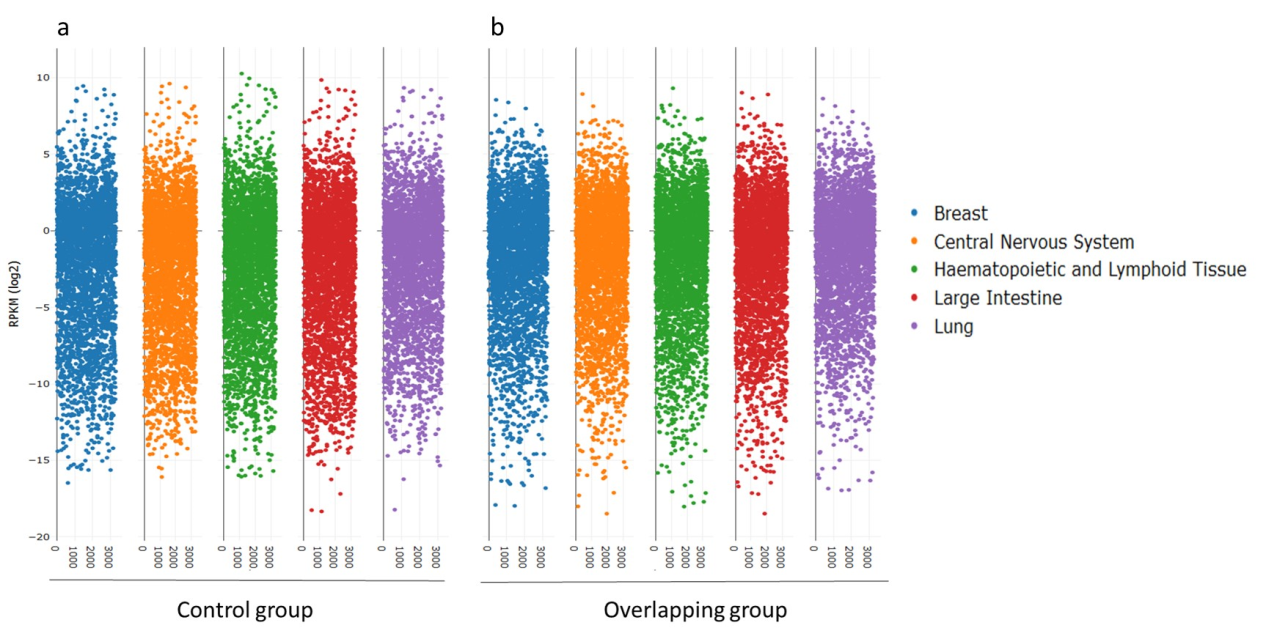
**

**Supplementary Figure 5.** Expression distribution of non-overlapping genes and paired overlapping genes in different tissues.

RPKM (log2) distributions of (a) non-overlapping gene group and (b) paired overlapping gene group in five major tissues are illustrated in scatter plots. The five tissues are breast, haematopoietic and lymphoid tissue, central nervous system, large intestine, and lung.

**
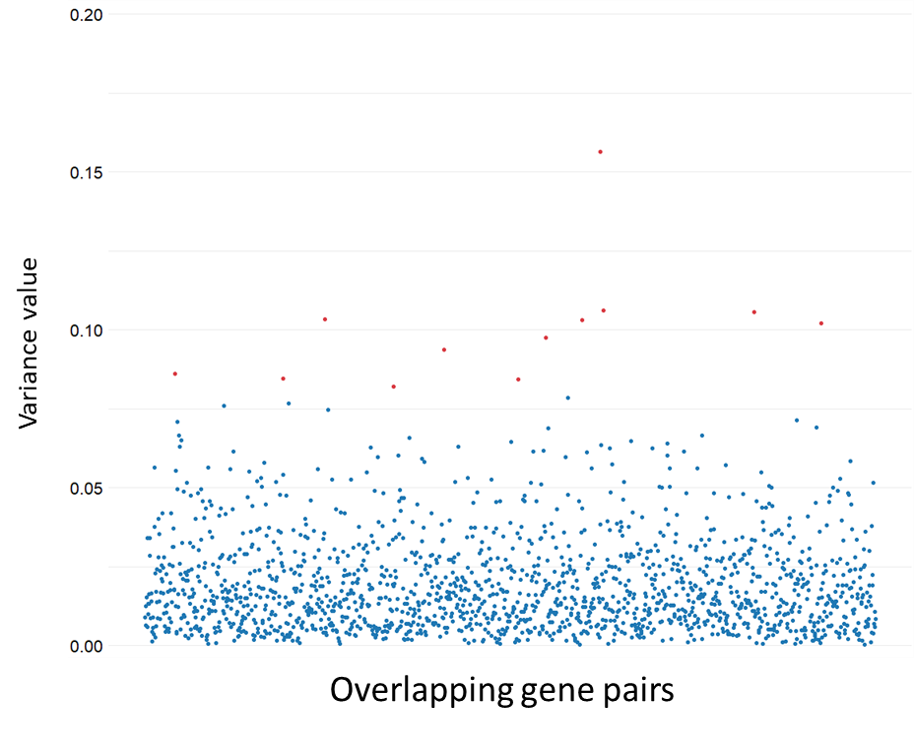
**

**Supplementary Figure 6.** Tissue variance distribution of paired overlapping genes.

This graph shows the variance values of overlapping protein-coding gene expression in five tissues. The gene pair variances which greater than 3 times of the interquartile range (IQR) are presented in red (12 gene pairs).
